# Molecular Dereplication and In Vitro and In Silico Pharmacological Evaluation of *Coriandrum sativum* against Neuroblastoma Cells

**DOI:** 10.3390/molecules27175389

**Published:** 2022-08-24

**Authors:** Maria Cristina Marcucci, Carlos Rocha Oliveira, Daniel Spindola, Alyne A. Antunes, Leila Y. K. Santana, Victor Cavalaro, Isabelle B. Costa, Ana C. de Carvalho, Thiago A. M. Veiga, Livia S. Medeiros, Lucas dos Santos Zamarioli, Carolina P. Gonçalves, Milena F. Santos, Simone S. Grecco, Vanessa Y. Suzuki, Lydia Masako Ferreira, Daniel M. Garcia

**Affiliations:** 1 Instituto de Ciência e Tecnologia, Universidade Estadual Paulista-UNESP, São José dos Campos 12231-280, SP, Brazil; 2 Grupo de Fitocomplexos e Sinalização Celular, Escola de Ciências da Saúde, Universidade Anhembi Morumbi, São Paulo 09972-270, SP, Brazil; 3 GAP Biotech, São José dos Campos 12231-280, SP, Brazil; 4 Programa de Pós Graduação em Engenharia Biomédica, Universidade Federal de São Paulo, São José dos Campos 12231-280, SP, Brazil; 5 Departamento de Química, Universidade Federal de São Paulo, Diadema 09920-000, SP, Brazil; 6 Mestrado Profissional em Farmácia, Universidade Anhanguera de São Paulo, São Paulo 09972-270, SP, Brazil; 7 Triplet Biotechnology Solutions, Bauru 17033-360, SP, Brazil; 8 Programa de Pós Graduação em Cirurgia Translacional e Disciplina de Cirurgia Plástica, Escola Paulista de Medicina (EPM), Universidade Federal de São Paulo, São Paulo 09972-270, SP, Brazil

**Keywords:** *Coriandrum sativum*, isocoumarins, neuroblastoma, apoptosis, cell death

## Abstract

The aim of this study was to investigate the cytotoxic activity of the *Coriandrum sativum* (*C. sativum*) ethanolic extract (CSEE) in neuroblastoma cells, chemically characterize the compounds present in the CSEE, and predict the molecular interactions and properties of ADME. Thus, after obtaining the CSEE and performing its chemical characterization through dereplication methods using UPLC/DAD-ESI/HRMS/MS, PM6 methods and the SwissADME drug design platform were used in order to predict molecular interactions and ADME properties. The CSEE was tested for 24 h in neuroblastoma cells to the establishment of the IC50 dose. Then, the cell death was evaluated, using annexin-PI, as well as the activity of the effector caspase 3, and the protein and mRNA levels of Bax and Bcl-2 were analyzed by ELISA and RT-PCR, respectively. By UHPLC/DAD/HRMS-MS/MS analysis, the CSEE showed a high content of isocoumarins-dihydrocoriandrin, coriandrin, and coriandrones A and B, as well as nitrogenated compounds (adenine, adenosine, and tryptophan). Flavonoids (apigenin, hyperoside, and rutin), phospholipids (PAF C-16 and LysoPC (16:0)), and acylglicerol were also identified in lower amount as important compounds with antioxidant activity. The in silico approach results showed that the compounds 1 to 6, which are found mostly in the *C. sativum* extract, obey the “Five Rules” of Lipinski, suggesting a good pharmacokinetic activity of these compounds when administered orally. The IC50 dose of CSEE (20 µg/mL) inhibited cell proliferation and promoted cell death by the accumulation of cleaved caspase-3 and the externalization of phosphatidylserine. Furthermore, CSEE decreased Bcl-2 and increased Bax, both protein and mRNA levels, suggesting an apoptotic mechanism. CSEE presents cytotoxic effects, promoting cell death. In addition to the promising results predicted through the in silico approach for all compounds, the compound 6 showed the best results in relation to stability due to its GAP value.

## 1. Introduction

Plants as a whole or referring to some of their main components have substantial protective effects on human carcinogenesis and mutagenesis [1]. Coriander (*C. sativum*) is one of this plants that has been studied for many years. The present literature supports the potential of coriander as a medicinal tree [2].

*C. sativum* belongs to *Apiaceae* family, cultivated mostly for seeds, which have a lemony citrus flavor and since antiquity, has been used in folk medicine [3].

Some coriander properties include antimutagenic and anticarcinogenic effects, as well as possible medicinal attributes including antispasmodic, carminative, stomachic, antidiabetic, and insomnia properties [4,5,6]. Furthermore, coriander bioactive compounds have the potential for a range of biological activities, including neuroprotective, anticonvulsant, anxiolytic, analgesic, hypolipidemic, hypotensive, and antimicrobial action [7,8]. Mahleyuddin et al. (2021) in a review, reported that different types of bioactive compounds such as alkaloids, essential oils, fatty acids, flavonoids, phenolics, reducing sugars, sterols, tannins, and terpenoids were extracted from *C. sativum*. Among them, higher concentrations were abundantly in the leaves through the presence of folates, ascorbic acid, gallic acid, caffeic acid, ferulic acid, and chlorogenic acid [9].

Regarding chemopreventive effects, *C. sativum* it induced apoptosis of cancer cells via death receptor and mitochondrial apoptotic pathways as demonstrated by increased caspase-8, -9, and -3 activities. The hypothesis is that the cascade is initiated by an activation of caspase-8, which leads to a sequential activation of caspase-9 and -3, respectively. Ascorbic acid, one of the bioactive compounds in *C. sativum*, has also been cited as chemopreventive through its cytotoxic activities against cancer cell lines [7].

Furthermore, *C. sativum* shows potential to prevent oxidative stress-related diseases and would be useful as supplement in combination with conventional drugs to enhance the treatment of diseases such as cancer [10].

Antioxidant activity prevents the formation of free radicals, decreasing oxidative stress, and avoiding destruction of essential molecules like proteins, DNA, and lipids [11]. That antioxidant activity showed a neuroprotective activity in Alzheimer’s disease models and other models with focus in tumoral proliferative glia and neurons models [4,12].

In this study, our goals were to evaluate the antiproliferative effects of crude extract of *C. sativum*, using the neuroblastoma cell line (SH-SY5Y), chemically characterize compounds present in the crude extract by means of de-replication using UPLC/DAD-ESI/HRMS/MS, and finally, to predict molecular interactions and properties of ADME, using PM6 methods and the SwissADME drug design platform.

## 2. Results

### 2.1. Phytochemical Analysis of the C. sativum Ethanolic Extract (CSEE)

There is a great variety of flavonoids and phenolic acids in the constitution of the secondary metabolism of the coriander [13], being the phenolic compounds attributed the responsibility for the antioxidant activity [14]. Antioxidants refer to any substances in attendance at stumpy concentration in foodstuffs and capable of significantly averting oxidation by playing a responsibility in antioxidation as a free radical scavenger [15].

The phytochemical prospecting tests, which aimed to characterize the main compounds of the secondary metabolism of the plant species, besides characterizing it botanically, were performed according to the proposed methodologies and the results are found in Table 1.

**Figure 1:**
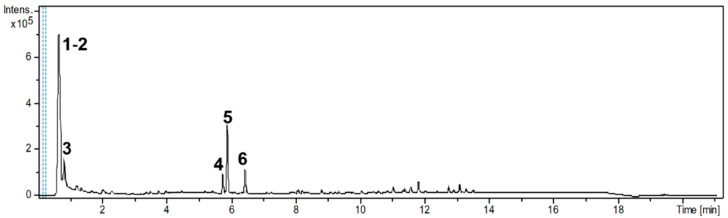
UV chromatogram of CSEE obtained by UPLC/DAD-ESI/HRMS/MS analysis (λ 190–800 nm).

**Figure 2:**
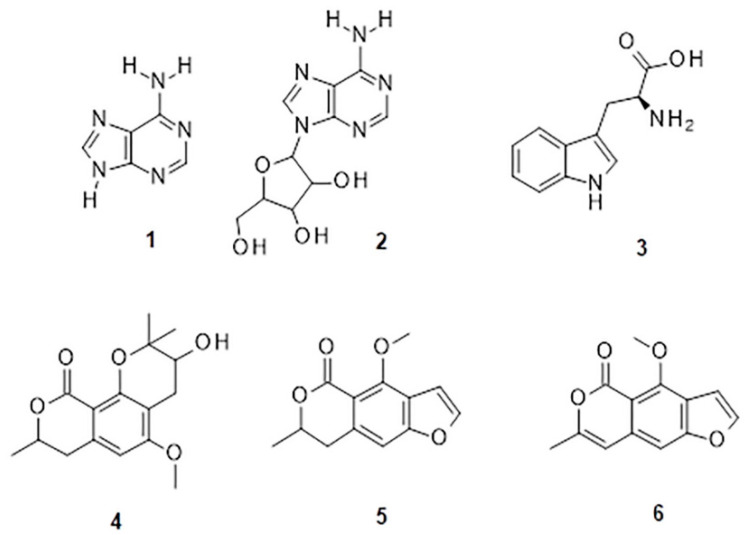
Chemical structures of major compounds identified from CSEE by mass spectrometry.

The UPLC/DAD-ESI/HRMS/MS analysis indicated five major peaks at UV chromatogram (Figure 1). In complement, the MS spectra of each peak allowed for the identification of six major compounds (**1**–**6**), whose structures are presented at Figure 2. The nitrogen compounds—adenine (**1**), adenosine (**2**), and tryptophan (**3**), and the isocoumarins— coriandrone B (**4**), dihydrocoriandrin (**5**), and coriandrin (**6**).

For **1**, it was observed the ion with *m*/*z* 136.0614 [M + H]^+^ (C_5_H_6_O_5_, calculated for 136.0618, Δ = 2.4 ppm); while for **2**, it was observed the ion with *m*/*z* 268.1044 [M + H]^+^ (C_10_H_14_N_5_O_4_, calculated for 268.1040, Δ = −1.5 ppm), and at the MS^2^ spectra of **2**, it was observed as a fragment ion *m*/*z* 136.0611 [M + H]^+^ that was attributed to adenine, which allowed for the confirmation of **2** as an adenine derivative. For **3**, it was observed an ion with *m*/*z* 205.0965 [M + H]^+^ (C_11_H_13_N_2_O_2_, calculated for 205.0972, Δ = 2.6 ppm), and the MS^2^ spectra of **3** displayed a fragment ion at *m*/*z* 91.0550 [M + H] ^+^ that could be attributed to the dissociation between the amino acid moiety and its side chain. For **4**, it was observed an ion with *m*/*z* 293.1400 [M + H]^+^ (C_16_H_21_O_5_, calculated for 239.1384, Δ = −5.8 ppm), the MS^2^ spectra of **4** displayed fragment ions at *m*/*z* 203.0718 [M + H]^+^, *m*/*z* 233.0833 [M + H]^+^, *m*/*z* 187.0759 [M + H]^+^, *m*/*z* 173.0600 [M + H]^+^, and *m*/*z* 161.060, which also could be observed and confirmed through the comparison with the coriandrone B predicted spectra [16]. The presence of fragment ion *m*/*z* 233.0833 could be a result of the tetrahydropyran opening, which allowed for the distinction between Coriandrones A and B [16]. The isofuranocoumarins **5** and **6** presented ions with *m*/*z* 233.0819 [M + H]^+^ (C_13_H_13_O_4_, calculated for 233.0808, Δ = −4.9 ppm) for **5**, while for **6**, an adduct ion with *m*/*z* 253.0475 [M + Na]^+^ (C_13_H_10_O_4_Na, calculated for 239.1384, Δ = −1.0 ppm) was observed. The MS^2^ spectra of **5** displayed fragment ions at *m*/*z* 215.0890 [M + H]^+^, *m*/*z* 187.0744 [M + H]^+^, and *m*/*z* 159.0890 [M + H]^+^. The fragment ions at *m*/*z* 187.0744 [M + H]^+^ and *m*/z 159.0890 [M + H]^+^ could be associated with the lactone ring opening. Both fragments also could be observed through the comparison with the dihydrocoriandrin predicted spectrum [16]. The structural identification of **6** was possible due to the presence of the fragment ions at *m*/*z* 189.0512 [M + H]^+^ and 131.0852 [M + H]^+^, which corroborates with the fragments found in the predicted spectrum of coriandrin [16].

The GNPS database identified, through comparison of MS^2^ spectra with internal library, rutin (R_t_-2.3 min) at *m*/*z* 611.1596 [M + H]^+^ (C_27_H_31_O_16_, calculated for 611.1607, Δ = 1.8 ppm), (9c,12c,15c)-monolinolenin *m*/*z* 353.2686 [M + H]^+^ (C_21_H_37_O_4_, calculated for 353.2686, Δ = 0 ppm), Lyso-PC(16:0) *m*/*z* 496.3390 [M + H]+ (C_24_H_51_NO_7_P, calculated for 496.3398, Δ = 1.5 ppm), and PAF C-16 *m*/*z* 524.3679 [M + H]^+^ (C_26_H_55_NO_7_P, calculated for 524.3711, Δ = 6.0 ppm). Additionally, some minor compounds were identified, the isomer of **4**-coriandrone A, and the flavonoids quercetin, hyperoside, and apigenin. All MS data are summarized at Table 2.

### 2.2. Molecular Interactions and ADME Properties Predictions

**Figure 3:**
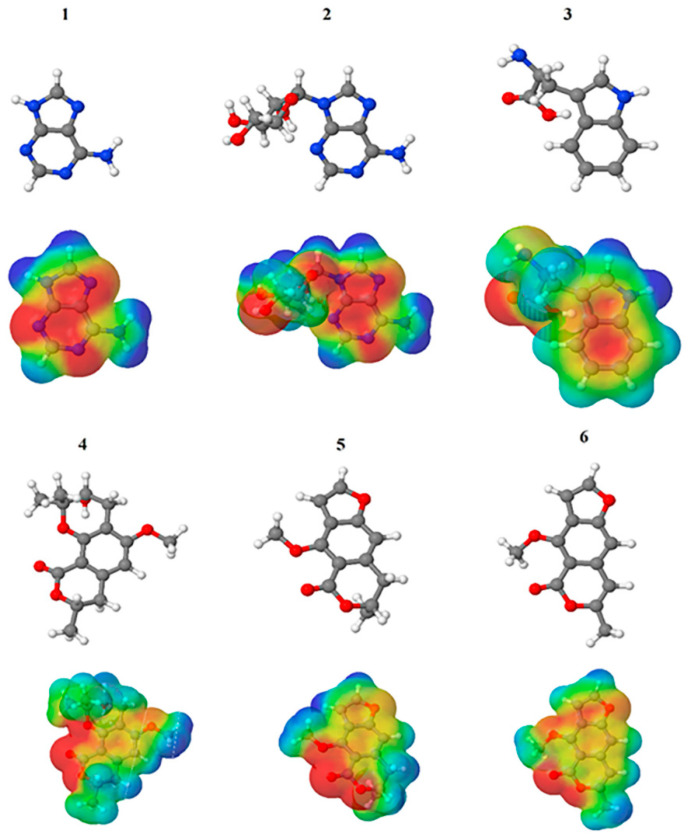
Electrostatic potential maps calculated onto the Connolly surfaces in the range of −0.1 to 0.1 obtained for compounds **1**–**6**.

The Molecular Electrostatic Potential Map (MEP) is a classic tool in the analysis of chemical activity, especially in the drug designing. In most cases, MEP is used as a qualitative approach. The MPE is based on the calculated properties of the charge density directly from the function of the molecular wave and measures the interaction of a positively charged point with the nuclei and electrons of a molecule. The interaction between the molecules occurs between regions of opposite electrostatic potentials. The improved accuracy of PM6 is particularly valuable for generating electronic descriptors for qualitative structure activity relationships (QSAR). When comparing the maps of electrostatic potentials of compounds **1**, **2**, and **3** (Figure 3) found mostly in coriander, we can see the presence of ribose in compound **2** in terms of volume, as adenosine creates an important increase in the total volume when we compare it with the compounds 1 and **3**. The negative charge concentration of compounds **1** and **2** (red region of the map) is very similar when we observe the pyrimidine and imidazole rings; however, the ribose found in compound **2** also has a high electronic density. Regarding compound **3**, tryptophan, the negative charge concentration in the indole ring, and the higher electron density is present on the carbonyl of the carboxylic acid group. In addition, compound **3** has large differences in shape and volume. Regarding the electrostatic potential maps obtained for compounds **4**, **5**, and **6**, it is possible to observe a small variation in relation to the shape and volume. The negative charge distribution is also relatively similar, mainly between compounds **4** and **5**, where it is predominant over the carbonyl of isochron-1-one.

The energies of the frontier orbitals, that is, the highest occupied molecular orbital (HOMO) and the lowest unoccupied molecular orbital (LUMO), were also calculated. These results are quantitative descriptive and are widely used, which play an important role in the chemistry reaction and in the formation of several charge complexes. The energy of HOMO is directly related to the potential ionization of the compound and the ability of the molecule to create nucleophiles. The energy of LUMO is directly related to electronic affinity, characterized by the susceptibility of the compound to nucleophiles in relation to them. The difference between the energies of the HOMO-LUMO orbitals, called GAP, is an important indicator of molecular stability. Molecules with a low GAP value are generally reactive, while molecules with a high GAP value indicate high stability and low possibility to react with another compounds [17]. Table 3 presents the values calculated for the HOMO and LUMO orbitals of compounds **1**–**6**, as well as the GAP value. The electronic density of frontier orbitals is a useful way to the detailed characterization of donor–acceptor interactions and the most of chemistry reactions occur at the location of the highest electronic density in the frontier orbitals.

Considering that the higher the energy of HOMO, the greater the electron-donor capacity, and that the lower the energy of LUMO, the lower the resistance to accept electrons, we can define that among the proposed new compounds, compound **6** has the highest GAP value and may have high molecular stability. Compound **2**, which has the lowest GAP value, can be considered the least stable compound. Thus, it is possible to predict that compound **6** may demonstrate greater biological activity; however, this is not an indicator of low reactivity for other compounds. The pharmacokinetic properties of the compounds were evaluated, and the SwissADME toolbar was employed. The Swiss ADMET Predictor is a designed software to estimating pharmacokinetic parameters/properties of drug-like compounds from their molecular structures [18]. Lipinski proposed four ADMET properties called the “Rule of Five”. This rule of five was the authentic and “most well-known rule-based filter” of drug-likeness, which is used to examine if the compound can be well absorbed orally or not. The rule of five includes: molecular weight (MW) ≤ 500; octanol/water partition coefficient (iLOGP) ≤ 5; number of hydrogen bond donors (HBDs) ≤ 5 (accounted in function of NH or OH groups in the molecule); and number of hydrogen bond acceptors (HBAs) ≤ 10 (accounted in function of N or O atoms in the molecule). Under the Rule of Five, a molecule can only be orally active/absorb if it does not violate any two or more rules [19]. Table 4 represented some of the ADMET properties/parameters for the compounds **1**–**6**.

Physicochemical properties calculated on SwissADME: MW: molecular weight; HBDs: hydrogen bonding donor; HBAs: hydrogen bonding acceptor; iLogP: octanol/water partition coefficient; nV: number of violations; TPSA: total polar surface area; LogS: coefficient of solubility determined by the ESOL method; Class: insoluble < –10 < poor < –6 < moderately < –4 < soluble < –2 < very < 0 < highly; %ABS was expressed by the equation %ABS = 109 – (0.345 × TPSA).

According to the results obtained through the theoretical studies, all compounds obey the standards of the Lipinski Rule, thus indicating a good oral bioavailability during the administration of this substance when in an oral pharmaceutical form. The percentage of absorption showed results for all compounds between 60.85 and 92.20%, indicating that these compounds have a good permeability in the cellular plasmatic membrane. The LogS of many substances already approved for use in therapeutics have a value greater than −4.00; in this study the compounds showed a value between −0.68 and 3.44, and the compounds **1**, **2**, **3**, and **4** were considered soluble, while compounds **5** and **6** were considered moderately soluble. The aqueous solubility of a compound significantly affects its absorption and distribution characteristics. Typically, a low solubility goes along with a bad absorption and, therefore, the general aim is to avoid poorly soluble compounds. The score of all compounds in relation to the drug score was determined by combining records of similarity with already approved drugs, as lipophilicity, solubility, molecular mass, and toxicity risks, and a single numeric value was given, which ranges from 0.0 to 1.0 and can be used to predict the global potential of a compound as a new drug candidate.

### 2.3. Antioxidant Activity

The free radical scavenging activity of the extracts were measured by the radical scavenging ability, using 1.1-diphenyl-2-picrylhydrazil (DPPH). The quantification of the sample was performed, with an EC_50_ of 27.27 ± 1.21 µg/mL.

### 2.4. CSEE Decreased the Viability of SH-SY5Y Cells, and Induces Apoptosis via the Intrinsic Pathway

**Figure 4:**
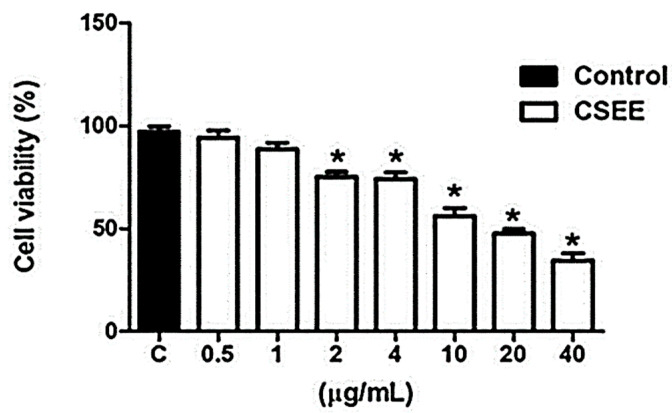
Effects of CSEE on SH-SY5Y cell line viability. Cells were treated with different concentrations of CSEE for 24 h, and the IC_50_ was defined as the study test concentration (20 μg/mL). Data shown are representative of three independent experiments. The values are expressed as mean ± SEM and * *p* < 0.05 indicates statistical difference (unpaired *t*-test).

In order to verify the cytotoxic activity of CSEE, the IC50 of the extract was initially determined (Figure 4). Thus, 20 μg/mL of CSEE was defined as the study concentration.

**Figure 5:**
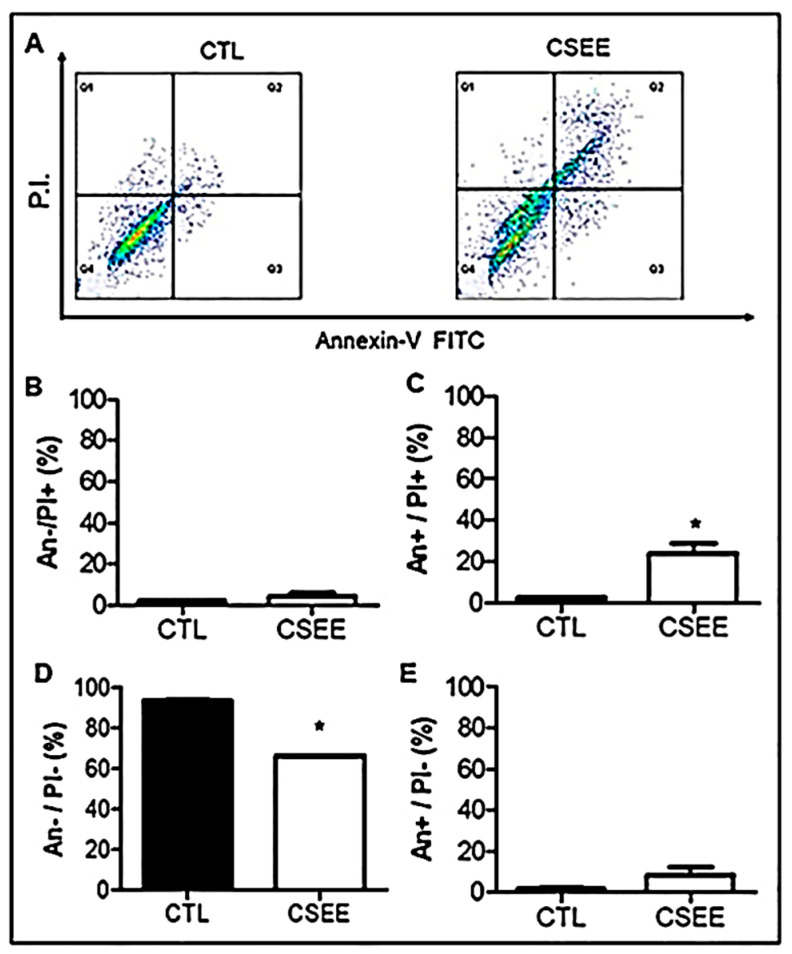
Effects of CSEE on phosphatidylserine externalization in cells SH-SY5Y. Annexin V-FITC/PI double staining. Cells were treated with the IC_50_ study test concentration (20 μg/mL) for 24 h. (**A**) Density plots of Annexin V-FITC/PI fluorescence. (**B**) Necrotic cells (AnnexinV−/PI+). (**C**) Late apoptotic/necrotic cells (AnnexinV+/PI+). (**D**) Viable cells (AnnexinV−/PI−). (**E**) Apoptotic cells (AnnexinV+/PI−). Data shown are representative of three independent experiments. The values are expressed as mean ± SEM and * *p* < 0.05 indicates statistical difference (unpaired *t*-test).

Aiming to identify cell death modalities induced by CSEE in SH-SY5Y cells were stained with FITC-labeled AnnexinV, which binds to PS, and PI, a small fluorescent dye that is excluded from live cells but stains the nuclei of cells with a ruptured or otherwise permeabilized plasma membrane. Hence, the combination of AnnexinV/PI cell staining was used to distinguish apoptotic and necrotic cell death (Figure 5A), CSEE (20 µg/mL, 24 h), decreased SH-SY5Y cell viability (Figure 5B), and increased the percentage of cells in early or late apoptosis after 24 h of incubation (Figure 5C,D, respectively). However, there was no significant increase in cell death by necrosis when compared with untreated cells (Figure 5E).

**Figure 6:**
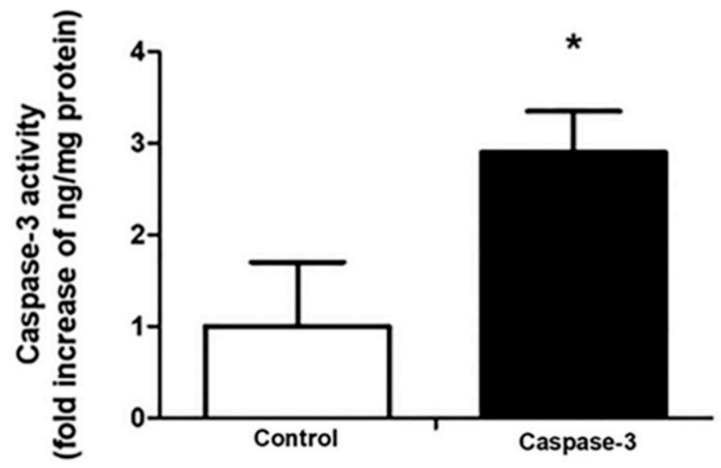
Effects of CSEE on caspase-3 activity. The SH-SY5Y cells were treated with 20 µg/mL of CSEE for 24 h. The results showed significant increase in caspase-3 activity on CSEE-treated cells vs. control group (non-treated cells) * *p* < 0.05. Data shown are representative of three independent experiments. The values are expressed as mean ± SEM.

An evaluation of the effects of CSEE treatment on the caspase-3 activity was determined and it was possible to observe that CSEE (20 µg/mL) induced a significant regulation of cleaved caspase-3 in treated samples in almost half of the treated cells (Figure 6), which strongly suggests apoptosis induction properties for CSEE.

### 2.5. Bcl-2 and Bax Expression in SH-SY5Y Cells

**Figure 7:**
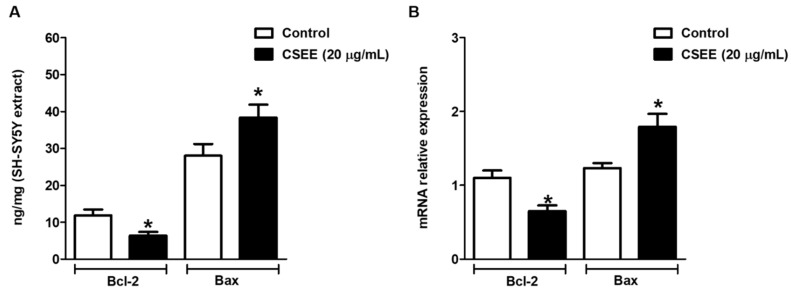
Effects of CSEE on Bax and Bcl2 expression. (**A**) CSEE reduces Bcl-2 protein level and increases the Bax protein level of SH-SY5Y cells (* *p* < 0.05). (**B**) CSSE reduces Bcl-2 mRNA levels and increases mRNA levels of SH-SY5Y cells (* *p* < 0.05). Values are expressed as mean ± SEM. SH-SY5Y cells were treated with 20 μg/mL of CSEE for 24 h. Data shown are representative of three independent experiments.

We investigated the relationship between Bcl-2 and Bax expression in SH-SY5Y cells. Figure 7A,B shows the effects of CSEE on the protein and mRNA levels of Bcl-2 and Bax. The protein and mRNA levels of Bcl-2, which promote anti-apoptosis effects, showed significant reduction in the CSEE-treated cells compared with the control group (non-treated cells); in contrast, the levels of Bax protein and mRNA, which promote pro-apoptosis effects, were increased in the CSEE-treated cells.

## 3. Discussion

The benefits of medicinal plants have been reported by literature as an important support to the quality of life of patients with cancer when used in association with conventional antineoplastic therapies [20]. Thus, the need for further research in order to investigate potential candidates for new therapeutic applications may start with its popular use [21].

Drug therapy has long known of the importance of screening secondary metabolites in the development of new drugs, and screenings already carried out show that the phytochemical profile of *C. sativum* indicates the presence of polyphenols, terpenes, reducing sugars, alkaloids, fatty acids, and sterols [22].

Numerous pharmacological effects are attributed to *C. sativum*, such as anxiolytic, antidepressant, sedative-hypnotic, anticonvulsant, neuroprotective, antibacterial, antifungal, anthelmintic, antioxidant, anti-inflammatory, analgesic, and antiproliferative effects [23].

In this study, we verified the presence of flavonoids and phenolic compounds in the extract of C. sativum, which was then confirmed by mass spectrometry.

Isocoumarins and nitrogen compounds were also found. Coriandrone A and B were isolated from *C. sativum* along with other isocoumarins (coriandrin and dihydrocoriandrin) and their structures, which were determined by spectroscopic means and X-ray crystallography [24]. Herein, the compounds identified at CSEE through UPLC/DAD-ESI/HRMS/MS analysis confirm these findings, evidencing the presence of Isofuranocoumarins-coriandrin and coriandrones A and B [25,26]. Besides that, studies have demonstrated the presence of these compounds in extracts of *C. sativum* leaves, especially the ethyl acetate fractions, in which the contents of dihydrocoriandrin (34.5%) and coriandrin (14.4%) were evaluated [27]. Products derived from *C. sativum*, as essential oil and extracts, exhibit antiproliferative [10,28,29] and antioxidant activities [30,31].

To verify the antioxidant activity of CSEE, an antioxidant activity test was performed using the DDPH method. The quantification of the sample was performed, with an EC_50_ of 27.27 ± 1.21 µg/mL [4], using the Supercrit Fluid Extraction (SFE, 300 bar, 40 °C) to extract *C. sativum* compounds, and obtained an EC_50_ 28.71 ± 2.41 µg/mL, similar to our results. However, the antioxidant activity of a methanolic extract and ethanol: water (80:20) from *C. sativum* was evaluated, with EC_50_ values of 2.2 ± 0.2 and 5.6 ± 0.6, respectively, which are much lower than ours. The authors reported that production procedures and climatic changes, such as average precipitation, harvesting time, altitude, and storage conditions, significantly influence the composition of phytochemicals in plants, directly reflecting on the antioxidant activity [31]. Some authors described that the antioxidant activity of *C. sativum* could be attributed to the action of (*E*)-2-decenal and linalool [32,33].

Confirming the CSEE antioxidant activity, assays were performed with SH-SY5Y cells in order to verify the ability of CSEE to cause cell death. Thus, after determining the IC_50_ (20 µg/mL), we verified that CSEE-induced apoptosis in SH-SY5Y cells, since the results showed that CSEE promoted greater phosphatidylserine externalization in treated SH-SY5Y cells (20 μg/mL), when compared to untreated cells.

The results obtained suggest that the likely mechanism involved in CSEE apoptotic induction is associated with the intrinsic pathway, where caspase-3 can be activated by a mitochondrial apoptotic pathway, contributing to cell apoptosis [34]. Our results showed that caspase-3 activity was significantly higher in treated SH-SY5Y cells compared to untreated cells. Exploring markers of the intrinsic apoptotic pathway, we observed a reduction in the levels of protein and mRNA of Bcl-2, an anti-apoptotic marker, and an increase in the levels of protein and mRNA Bax, a pro-apoptotic marker in SH -SY5Y cells treated with CSE. Data obtained in the literature describe the cytotoxic activity of *C. sativum* on SH-SY5Y cells [8,35], although the same cell line has been used to investigate the neuroprotective role of *C. sativum* [12]. Our results showed in vitro antitumor activity of CSEE, which may be associated with a cytotoxic effect of CSEE, due, at least in part, to the presence of isofurnacoumarins, choriandrin, and choriandrones A and B in CSEE, as demonstrated by UPLC/DAD-ESI /HRMS/MS. Our findings are in agreement with studies that showed antitumor activity of *C. sativum* against a variety of cells such as MCF-7 [8], K562 and HL60 leukemic cells [36], and HeLa cells [37].

According to the results obtained through the theoretical studies, all compounds indicated a good oral bioavailability during the administration in an oral pharmaceutical form. The score of all compounds was determined by combining records of similarity with already approved drugs, as lipophilicity, solubility, molecular mass, and toxicity risks, and a single numeric value was determined and can be used to predict the global potential of a compound as a new drug candidate.

Nevertheless, even though the in silico methods used in the development of new drugs are promising, it is important to emphasize their limitations, which can sometimes reveal up to 40% differences in pharmacokinetic parameters, such as area under the curve or maximum serum concentration, between observed and simulated data [38,39].

## 4. Material and Methods

### 4.1. Reagents

Dulbecco’s Modified Eagle’s Medium (DMEM) and all cell culture reagents were purchased from Life Technologies (CA, USA). Fetal bovine serum (FBS) and MTT were obtained from Himedia. FITC AnnexinV Apoptosis Detection Kit was bought from Becton Dickinson (NJ, USA). All other chemicals were of analytical grade. A colorimetric assay kit was used to measure caspase-3 activity (R&D Systems, Inc., Minneapolis, MN, USA). Enzyme-linked immunonosorbent assay (ELISA) kits were used to measure Bax and Bcl-2 protein levels (Elabscience, Houston, TX, USA). Ethanol solution (60%), for extraction procedures, was prepared using ethanol PA from Synth (Brazil). Sodium carbonate and aluminum chloride were obtained from Dinâmica (Brazil) and Synth, respectively, while Folin-Ciocalteu was obtained from Merck^®^. Gallic acid and quercetin standards were purchased from Sigma (Annapolis, MD, USA). Methanol was used for UPLC/MS procedures, acetonitrile and formic acid were HPLC grade from Merck^®^, and sodium formate was prepared from sodium hydroxide (Dinâmica, Brazil) and formic acid (Merck^®^).

### 4.2. Preparation of Plant Materials and Phytochemical Analysis

To obtain 500 g of totally dried plant, including roots, leaves, and stems, 3 kg of coriander were used, washed in running water, and dried in an oven between 30 °C and 37 °C for 6 days. After drying, the plant was processed in a knife mill and screened in mesh 80. In order to obtain the fluid *C. sativum* ethanolic extract-CSEE, 500 g of the dried and grinded plant was moistened with 100mL of 60% ethanol solution, i.e., in a proportion of 20% (*w*/*v*), for 48 h. After humidification the solution was transferred to a percolator, with 3 cm of hydrophilic cotton added to the bottom, together with more 100 mL of ethanol solution (60%). The macerate was added and lightly pressed. Filtration paper, porcelain chips, and 500 mL of 60% ethanol were added over the macerate, adjusting the tap to 5 drops/minute, as soon as the column of the macerate was impregnated with the extracting liquid. The percolation was maintained for 36 h, under controlled oven temperature. The fluid extract was subjected to evaporation at 45 °C, and at the end of 5 days of forced evaporation, the crude dry CSEE was collected and subjected to trituration in a mortar and sieved in an 80-mesh sieve, then kept in a dry environment with packages of silica gel [40,41].

#### 4.2.1. Total Soluble Solids

Next, 5.0 mL of fluid CSEE was added to pre-calibrated beakers and was taken to oven to dry at 70 °C. After drying, it was placed in the desiccator for cooling and then being weighed. The amount of soluble solids in the extract was calculated as follows:

$${\%}_{\left(w/V\right)}^{soluble\;solids}=\;\frac{\left(w-b\right)}{Va}\;\times \;100$$

where:

*b* = weight of the beaker;

*w* = final mass of the extract, after drying;

*Va* = volume of 5 mL.

#### 4.2.2. Estimation of Total Phenolic Content

In a 3 mL vessel we added 200 µL of the CSEE in 1:10 dilution and then added 1400 µL of water. Then, 160 µL of the sodium carbonate solution at 20% and then 240 µL of Folin-Ciocalteu was added, and the sample was shaken for few seconds and incubated in the absence of light for 2 h at room temperature [42]. The sample was shaken for a few seconds again, and the absorbance was measured at a wavelength of 760 nm (Cary-50 spectrophotometer, Varian-Inc, CA, USA). The total amount of phenols (in mg/mL) was calculated by a standard curve prepared with gallic acid in the same conditions of the assay. The procedure was performed in triplicate.

#### 4.2.3. Flavonoids

The CSEE was diluted 1:5 in methanol, and then 100 µL was transferred to the appropriate vessel and diluted in 1500 µL of methanol. Then, 400 µL of aluminum chloride was added in a concentration of 5% and stirred for a few seconds. The solution was incubated for 30 min in the absence of light at room temperature. The sample was agitated again for a few seconds and the absorbance of the sample was measured at wavelength of 425 nm [43,44]. The amount of flavonoids (in mg/mL) was calculated by a standard curve prepared with quercetin in the same conditions of the assay. The procedure was performed in triplicate.

### 4.3. Chemical Composition—Molecular Dereplication (UPLC/DAD-ESI/HRMS/MS)

First, 1.0 mg of CSEE was dissolved in 1 mL of methanol (HPLC grade), filtered at a 0.45 μm modified Polytetrafluoroethylene (PTFE) filter—Millex (Merck–Millipore), and subjected to a UHPLC/DAD-ESI/HRM/SMS analysis, according to the previously described protocol by de Carvalho [45]. A Shimadzu chromatographic system was used with a Kinetex 2.6 μ, C_18_ column, as the stationary phase, and kept in a column oven at 55 °C. Acetonitrile and ultrapure water, both with 0.1% of formic acid, were used as the mobile phase. The analytical conditions started from an exploratory gradient with a low concentration of strong elution solvent, 15% (Acetonitrile—ACN) to 95% of this same solvent for 12 min, and then the ACN concentration was kept constant for 4 min, returning the initial concentration after 1 min, and kept constant for 4 min. The UPLC effluent was subjected to electrospray ionization (ESI) and analyzed in a positive mode in high-resolution mass spectrometer, equipped with a QToF mass analyzer (Bruker Daltonics, Billerica, MA, EUA). The temperature of the drying gas was defined as 200 °C at a flow of 9 L min^−1^, 2 bar for the pressure of the nebulizer, and 4500 V of capillary voltage (kV). For the characterization of the compounds detected by UHPLC/DAD-ESI/HRMS/MS, fragments from well-resolved chromatographic bands were selected, with a mass-charge ratio (*m*/*z*) between 50 and 1200 Da. Sodium formate was used as a calibrator. The data were treated using the DataAnalysis 4.4 software (Bruker), and Extracted Ion Chromatograms (EIC) were generated using the Target Analysis 1.3 software as well as an “in house” list of target candidates, within the compound name and molecular formula, according to *C. sativum* chemical composition literature and “Coriander Genomics Database” [46,47]. For MS/MS spectra confirmation, fragmentation profiles of identified compounds were compared with those available at FOODB database (https://foodb.ca/, accessed on 20 July 2022) and with predicted mass spectra [48]. Additionally, the Global Natural Products Social Molecular Network (GNPS—open access platform) was used. The data were filtered by removing all MS/MS fragment ions within ±17 Da of the precursor *m*/*z*. MS/MS spectra were window filtered by choosing only the top 6 fragment ions in the ±50 Da window throughout the spectrum. The precursor ion mass tolerance was set to 0.01 Da and a MS/MS fragment ion tolerance of 0.02 Da. A network was then created where edges were filtered to have a cosine score above 0.6 and more than 5 matched peaks. The spectra in the network were then searched against GNPS’ spectral libraries. The library spectra were filtered in the same manner as the input data. All matches kept between network spectra and library spectra were required to have a score above 0.7 and at least 6 matched peaks [16,46].

### 4.4. Molecular Interactions and ADME Properties Prediction

Structural optimization was performed by the PM6 methods implemented in the semi-empirical quantum chemistry MOPAC 2016 software package. The files containing the three-dimensional information of each structure as well as the charge information of electrostatic potential were the input files viewed by the JMol software. The frontier orbital energy was calculated by the Chem3D Ultra 8.0. Data of orbital energies, dipole moment partial charges, and atomic volumes were used in the comparative analysis by which it was intended to carry out qualitative studies of chemical structure and biological activity (SAR). To evaluate the pharmacokinetic properties of the designed compounds, the 2D structure of the compounds were drawn on Chemdraw Ultra 12.0. Each structure was imported, and the structure smiley was entered at the interface of the website (http://swissadme.ch/, accessed on 20 July 2022). The SwissADME drug design study was run and the ADME properties/parameters were generated [18].

### 4.5. Antioxidant Activity

The activity of CSEE against free radicals was performed using DPPH (1,1-diphenyl-2-picrylhydrazyl radical, Sigma, USA). The extract was diluted so that the concentration of soluble solids was equal to 0.1%, and free radical decay curve was performed, where the initial absorbance was approximately 0.7300. The curve was made with eleven points, with a gradual increase of 40 µL of 0.1% extract (0; 40; 80; 120; 160; 200; 240; 280; 320; 360; 400 μL), completed with ethanol (q.s.1000 μL) that is, the first point had 1000 µL of ethanol and zero µL of extract, the second, 960µL of ethanol and 40µL of extract, and so on. After the solutions were prepared, 1000µL of DPPH (200 µM) was added to each of them, with a delay of 1 min per vessel and incubated for 30 min at room temperature in the absence of light. The absorbance decay was measured at 517nm, and the EC_50_ (concentration that eliminates 50% of free radical) was calculated by the ratio of the weighted least squares [16,49].

### 4.6. Cell Line and MTT Assay

SH-SY5Y human neuroblastoma cell line was obtained from the Rio de Janeiro Cell Bank, Rio de Janeiro, RJ, Brazil. The cell line was cultured in Dulbecco’s Modified Eagle’s Medium (DMEM) (Sigma Chemical Co, MD) supplemented with 10% fetal bovine serum, 2 mmol L^−1^ L-glutamine, 100 UI/mL penicillin and 100 µg/mL streptomycin, and 1 mg/mL fungizone in a humidified atmosphere at 37 °C in 5% CO_2_. The cells used in these experiments were of passage number 3–10. Cell viability was measured using the standard methylthiazol tetrazolium (MTT) assay, as previously described by Mosmmann (1983) [50]. The SH-SY5Y cell line was chosen for the study because it is an in vitro model widely used in studies related to neurotoxicity, oxidative stress, and neurodegenerative diseases [51]. To measure cell viability, SH-SY5Y cells were seeded at a density of 5 × 10^3^ cells/well and treated with different concentrations of CSEE (0.5; 1.0; 2.0; 4.0; 10.0; 20.0; and 40.0 µg/mL) for 24 h. Next, 10 μL of a 5mg/mL MTT solution (Sigma-Aldrich) were added to each well. After 4 h, the samples were reincubated with 100µL dimethyl sulfoxide (DMSO), and then optical density was measured in a FlexStation^®^ 3 multimode Benchtop Reader (Molecular Devices, CA, USA) at 540 nm. The IC_50_ value was calculated using the GraphPad Prism^TM^ v5.01 (GraphPad Software, Inc., San Diego, CA, USA).

### 4.7. Cell Death Evaluation by Annexin V/FITC–PI Staining and Flow Cytometry Analysis

To identify CSEE-induced cell death modalities in SH-SY5Y cells, we analyzed phosphatidylserine (PS) exposure in apoptotic cell membrane, a hallmark of apoptosis. Thus, SH-SY5Y cells were stained with FITC-labeled Annexin V, which binds to PS, and propidium iodide (PI), a fluorescent dye that is incorporated into the DNA of a ruptured or permeabilized plasma membrane [52,53]. Following 24 h of CSEE treatment, SH-SY5Y cells were stained with fluorescein isothiocyanate (FITC)-conjugated Annexin V and PI according to the manufacturer’s instructions (Annexin V/FITC Apoptosis Detection Kit, BD Pharmingen, CA, USA). AnnexinV/PI (viable cells), AnnexinV+/PI (apoptotic cells), AnnexinV/PI+ (necrotic cells), and AnnexinV+/PI+ (secondary necrosis/late apoptosis) populations were evaluated by flow cytometry. 10^4^ events were collected for each sample in a FACSCalibur flow cytometer (Becton-Dickinson, Mountain View, CA, USA). Sample data were acquired with CellQuest software (Becton-Dickinson) and analyzed using FlowJo^TM^ v10.0 (Tree Star, Inc., Ashland, OR, USA).

### 4.8. Caspase-3 Activity Measurement

A colorimetric assay kit was used to measure caspase-3 activity. The assay was conducted according to the protocol provided by the manufacturer (R&D Systems, Inc., Minneapolis, MN, USA). Briefly, SH-SY5Y cells were lysed in lysis buffer (20 mM HEPES; 1 mM EDTA; 1 mM EGTA; 50 mM NaF; 10 mM β-glycerophosphate; 2 mM MgCl2; 150 mM NaCl; 10 mM KCl, 1% NP-40; 1 mM DTT; 1 mM benzamide; 1 mM PMSF; 10 μg/mL of aprotitin, leupeptin, and pepstatin A). Then, proteins (150 μg) were incubated with caspase-3 substrate, Ac-DEVD-pNA-(7-amino-4 methyl coumarin) at 37 °C for 1~2 h, and the absorbance was measured at 405 nm. A recombinant caspase-3 enzyme (R&D Systems, Inc., Minneapolis, MN, USA) was used for generating a standard and caspase-3 activity was calculated as ng/mg protein. The data were expressed as a fold increase in activity.

### 4.9. ELISA (Enzyme Linked ImmunonoSorbent Assay)

Bax and Bcl-2 protein levels in SH-SY5Y cells were measured using ELISA kit Bax (Elabscience, Houston, USA) and Bcl-2 (Elabscience, Houston, TX, USA), in accordance with the manufacturer’s protocols. Basically, after treatment of SH-SY5Y cells with CSEE (20 μg/mL), samples were added to plates pre-coated with antibodies to Bcl-2 or Bax. After incubation, biotinylated antibodies to Bcl-2 or Bax and avidin horseradish peroxidase conjugate were added and incubated, followed by the addition of the substrate. After incubation, the enzyme-substrate reaction was stopped, followed by spectrophotometric determination (OD450nm), and the values were converted to protein level according to the standard curve.

### 4.10. Reverse Transcription-Quantitative PCR (RT-qPCR)

After treatment with 20 µg/mL of CSEE, total RNA extracted from SH-SY5Y cells samples was converted to cDNA using a SuperScript^®^ III RT kit (Invitrogen, Carlsbad, CA, USA), according to the manufacturer’s protocol. The concentration of RNA was detected using a NanoDrop 2000 (Thermo Fisher Scientific, Inc., Waltham, MA, USA). GAPDH was used as the internal control. The thermocycling conditions were as follows: 95 °C for 10 min followed by 35 cycles of 95 °C for 15 s and 55 °C for 40 s. The 2-ΔΔCq method was used to quantify the relative gene expression levels of the target genes. Relative standard curves were generated by serial dilutions and all samples were run in triplicates. For PCR quantities analysis, the primers of Bcl-2, Bax, and GAPDH genes were designed using Allele ID software. Thus, the sense and anti-sense sequences of primers used in qRT-PCR analysis were: Bcl-2 Forward: ‘5-TTGTGGCCTTCTTTGAGTTCGGTG-3’; and Reverse: ‘5-GGTGCCGGTTCAGGTACTCAGTCA-3’; Bax Forward: ‘5-CTGTGCACCAAGGTGCCGGAACT-3’; and Reverse: ‘5-CACCCTGGTCTTGGATCCAGCCC-3’; GAPDH Forward: ‘5-ACCCAGAAGACTGTGGATGG-3’; and Reverse: ‘5- TCTAGACGGCAGGTCAGGTC-3’.

### 4.11. Statistical Analysis

For treatment of the statistical analysis of the data, the results were expressed as mean ± standard deviation, analyzed independently. The tests were analyzed by one-way ANOVA followed by application of the Tukey test using the program Graph Pad version 5.0. Significant differences were considered as *p* < 0.05.

## 5. Conclusions

In this study, we reported for the first time the potential of CSEE as an inducer of cell death in an in vitro cell model of neuroblastoma. The antiproliferative activity of CSEE may be associated with compounds such as coumarins present in the studied extract. Our results suggest a cytotoxic potential of CSEE, in addition to initial pharmacokinetic parameters, obtained by in silico method. Further studies are needed to elucidate the antiproliferative mechanism presented here in order to better understand the cytotoxic potential of *C. sativum*.

## Figures and Tables

**Table 1:** CSEE a phytochemical prospecting.

Test	g/100 mL	CV (%)
Soluble solids	0.487 ± 0.007	7.40
Total phenolic content	2.057 ± 0.017	0.82
Total flavonoid content	1.079 ± 0.013	1.21

CV% is variation coefficient: (standard deviation/average) * 100.

**Table 2:** Compounds identified at CSEE thought UPLC/DAD-ESI/HRMS/MS analysis.

RT [min]	UV-Vis [nm]	Compound Name (N) *	NMF *	*m*/*z*-Experimental [M + H]^+^	*m*/*z*-Calculated [M + H]^+^	Δ; Err [ppm]	mSigma	MS^2^ (%)
0.7	201; 229; 262	adenine **(1)**	C_5_H_5_N_5_	136.0614	136.0618	2.4	8.3	-
		adenosine **(2)**	C_10_H_13_N_5_O_4_	268.1044	268.1040	−1.5	23.1	136.0622 (100); 137.0607 (9.2); 119.0414 (5.3)
0.9	190	tryptophan **(3)**	C_11_H_12_N_2_O_2_	205.0966	205.0972	2.6	11.7	91.0550 (100)
2.3	190; 256; 303; 349	quercetin	C_15_H_10_O_7_	303.0508	303.0499	−2.7	10.2	-
		hyperoside	C_21_H_20_O_12_	465.1020	465.1028	1.6	35.3	86.0956 (100)
		Rutin	C_27_H_30_O_16_	611.1596	611.1607	1.8	31.1	303.0486 (100); 85.0283 (14.5)
3.8	190; 232	apigenin	C_15_H_10_O_5_	271.0606	271.0601	−1.9	32.8	243.9555 (100)
5.4	217.5	coriandrone A	C_16_H_20_O_5_	293.1391	293.1384	−2.4	5.2	203.0710 (100); 221.0808 (80.1); 173.0602 (51.4); 191.0693 (25.1)
5.8	225; 266; 307	coriandrone B **(4)**	C_16_H_20_O_5_	293.1400	239.1384	−5.8	4.1	203.0716 (100); 215.0698 (77.8); 187.0758 (58.7); 173.0598 (56.3)
5.9	229	dihydrocoriandrin **(5)**	C_13_H_12_O_4_	233.0820	233.0808	−4.9	18.1	129.0700 (100); 187.0742 (98.4); 172.0525 (76.7); 200.0466 (71.2); 159.0808 (40.1); 144.0581 (36.7)
6.5	190; 248; 286; 298; 343	coriandrin **(6)**	C_13_H_10_O_4_	253.0474 **	253.0471 **	−1.0	16.5	93.0721 (100); 81.0750 (71.2); 107.0799 (70.5)
8.4	190	monolinolenin	C_21_H_36_O_4_	353.2686	353.2686	0	2.3	93.0714 (100); 81.0689 (87.8); 107.0855 (68.1)
9.3	219	lyso-PC (16:0)	C_24_H_50_NO_7_P	496.3390	496.3398	1.5	5.4	184.0727 (100); 104.1080 (37.7); 86.0953 (36.6); 124.9994 (28.2);
10.3	219	PAF C16	C_26_H_54_NO_7_P	524.3679	524.3711	6.0	9.2	184.0731 (100); 104.1068 (39.2); 125.0011 (38.2); 86.0962 (29.1)

* Major compounds are also labeled by numbers from UV chromatogram; ** Sodium adduct ion of coriandrin [M + Na]^+^.

**Table 3:** Frontier orbitals calculated for compounds **1–6** and their GAP values.

Compound		HOMO (eV)	LUMO (eV)	GAP (eV) *
**1**	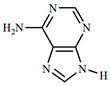	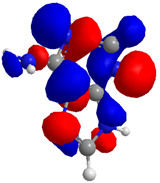	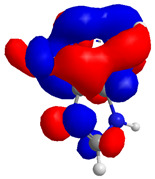	4.182218
		−8.246172	−4.063954	
**2**	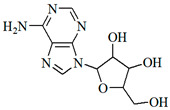	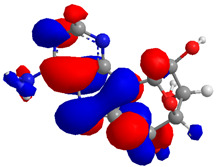	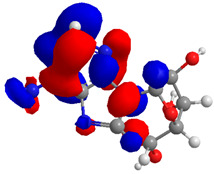	3.497648
		−6.777479	−3.279831	
**3**	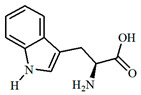	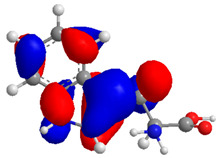	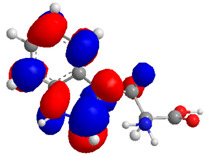	6.628958
		−10.133024	−3.504282	
**4**	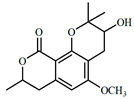	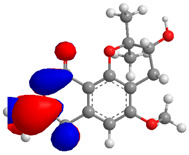	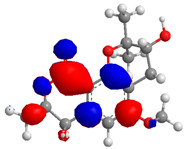	5.205865
		−8.710147	−3.504282	
**5**	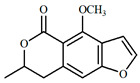	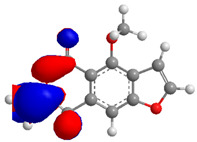	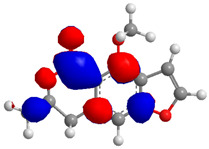	6.119369
		−8.725406	−2.606037	
**6**	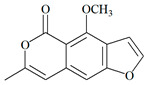	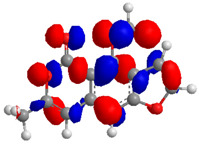	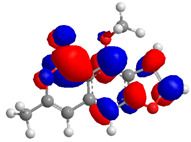	8.691302
		−11.434933	−2.743631	

* The GAP calculation was performed from the difference of the energy value of the LUMO orbital by the energy value of the HOMO orbital.

**Table 4:** ADMET parameters found for the compounds **1**–**6** using the free toolbar SwissADMET Predictor.

Compound	Lipinski Parameter					TPSA (Å²)	LogS	Class	%ABS (%)	Drug Score
	MW (g/mol)	HBDs	HBAs	iLOGP	nV					
**1**	135.13	2	3	0.42	0	80.48	−1.43	Soluble	81.23	0.55
**2**	267.24	4	7	0.41	0	139.54	−1.05	Soluble	60.85	0.55
**3**	204.23	3	3	0.99	0	79.11	−0.68	Soluble	81.70	0.55
**4**	292.33	1	5	2.71	0	64.99	−3.20	Soluble	86.57	0.55
**5**	232.23	0	4	2.33	0	48.67	−3.26	Moderate	92.20	0.55
**6**	230.22	0	4	2.43	0	52.58	−3.44	Moderate	90.85	0.55

## Data Availability

All raw data will be available upon a reasonable justification request.

## Sample Availability

Samples of the compounds will be available upon a reasonable justification request.
